# HLA Alleles and Haplotype Distribution Across Russian Population Groups

**DOI:** 10.3390/ijms27115063

**Published:** 2026-06-03

**Authors:** Varvara Kucherenko, Natalia Doroschuk, Elizaveta Sarygina, Olesya Sagaydak, Viktor Bogdanov, Olga Mityaeva, Julia Krupinova, Mary Woroncow, Eugene Albert, Pavel Volchkov

**Affiliations:** 1 Federal Research Center for Innovator and Emerging Biomedical and Pharmaceutical Technologies, 125315 Moscow, Russia; kucherenko_vv@academpharm.ru (V.K.); bogdanov_vp@academpharm.ru (V.B.); mityaeva_on@academpharm.ru (O.M.); krupinova_ua@academpharm.ru (J.K.); 2 Evogen LLC, 115191 Moscow, Russia; doroshchuk@evogenlab.ru (N.D.); sarygina@evogenlab.ru (E.S.); sagaydak@evogenlab.ru (O.S.); 3 National Medical Research Center of Cardiology Named After Academician E.I. Chazov, 21552 Moscow, Russia; 4 Moscow Center for Advanced Studies, 123592 Moscow, Russia; 5 Faculty of Medicine, Lomonosov Moscow State University, 119991 Moscow, Russia; innovations.endo.m@gmail.com

**Keywords:** HLA, allele frequency, WGS, Russia

## Abstract

Human leukocyte antigen (HLA) loci are highly polymorphic genome regions, with allele frequencies varying significantly across different populations. Population HLA frequency databases may contain biases and make cross-study comparison complicated due to varying data curation protocols, genotyping methodologies, resolution, and inconsistencies in the selection criteria for population samples. This study presents HLA allele frequencies of class I (HLA-A, -B, -C) and class II (HLA-DRB1, -DQB1, -DQA1), as well as their combined haplotypes obtained from over 18,000 whole genome sequencing samples of the Russian population. The cohort was stratified based on PCA and admixture components, providing frequencies for 14 different ethnic groups. For 12 groups cohort size allowed us to reach average saturation of 96% of allele frequencies in groups. Moreover, we demonstrated the utility of composed statistics for disease population study using type 1 diabetes (T1D) as an example. Genetically defined population clusters with similar aggregated genetic risk for T1D demonstrated substantial differences in frequencies of risk and protective HLA alleles. Obtained frequency data were made publicly available through the Allele Frequency Net Database improving previously sparse coverage in HLA frequencies data for the East Europe and North Asia regions.

## 1. Introduction

Human leukocyte antigen (HLA) diversity, while essential for immunity, creates a major challenge for donor-recipient matching in transplantation. Currently, the most efficient way to tackle the haplotype matching problem is the construction of a large, usually nation-wide register of HLA haplotypes. Efficient construction of such registries requires a good understanding of population-specific variations in HLA haplotype composition to ensure appropriate coverage for diverse ethnic groups [1]. Core clinical typing focuses on HLA-A, -B, -C, -DRB1, -DQB1, and -DPB1 mostly defining the transplantation outcome. Extension to -DRB3/4/5 and -DPA1 HLA typing helps reduce the risk of graft failure and allows effective immunosuppression management [2,3,4].

Population-specific HLA haplotype information is aggregated in databases such as Allele Frequency Net Database (AFND) [5], iHLA-net [6], and PGG.MHC [7]. AFND provides the most extensive datasets, yet its utility is frequently limited by inconsistencies and variable data quality. In contrast, iHLA-net offers high standards for the validation of HLA allele frequencies [8,9]. The database requires the submission of a standardized data format, a detailed description of the allele and haplotype frequency calculation methods, and the inclusion of HWE tests [8]. The database is constrained by a small sample size and limited region representation. Meanwhile, PGG.MHC predominantly focuses on East Asian populations; the Han Chinese population entry currently has a sample size of 44,523. Although the database has a methodological description of data processing, it has no explicit data quality stratification [7].

Russia’s territory encompasses a wide range of ethnicities, including ethnic groups from the Caucasus region, Finno-Ugric, indigenous Siberian, Slavic, and Turkic populations. This significant diversity makes the study of HLA variation in the region particularly valuable for both medical and anthropological research. However, comprehensive data on HLA allele and haplotype frequencies across Russia’s distinct ethnic groups remain limited primarily because of heterogeneity of data, small sample sizes, and low resolution. For instance, the iHLA-net records from the Russian region comprise only 3 entries derived from the International HLA & Immunogenetics Workshop, and access to these data is restricted and requires prior permission [10,11]. PGG.MHC includes several (16) Russian populations represented, primarily derived from the Simons Genome Diversity Project, with a median sample size of two individuals [7,12]. AFND has the most extensive population set from the Russian region (53) including Bashkirs, Buryats, Khanty, Mansi, Tatars, Tuvans, etc. Still, some of the ethnicities are underrepresented or entirely absent. For example, the median sample size for Russian AFND entries is only 80. Furthermore, Yakut HLA frequencies are not yet represented in the database.

This study investigates the distribution of HLA class I (HLA-A, -B, -C) and class II (HLA-DRB1, -DQB1, -DQA1) alleles and haplotypes across diverse Russian populations, focusing on key loci of clinical significance. Using whole-genome sequencing (WGS) data from Bashkirs, Chechens, Russians, Tatars, Yakuts, and other groups, we aim to expand current HLA databases by incorporating a large, uniformly processed cohort of high-quality samples. The work explores HLA diversity within and between populations and identifies novel alleles that can refine our understanding of genetic variation across Russia. Ultimately, this research seeks to advance knowledge of the country’s immunogenetic landscape and to support developments in precision medicine, transplantation registries, and population genetics.

## 2. Results

### 2.1. Ethnic Stratification of the Cohort Revealed 14 Distinct Clusters Showing Consistent Admixture Patterns

**Figure 1:**
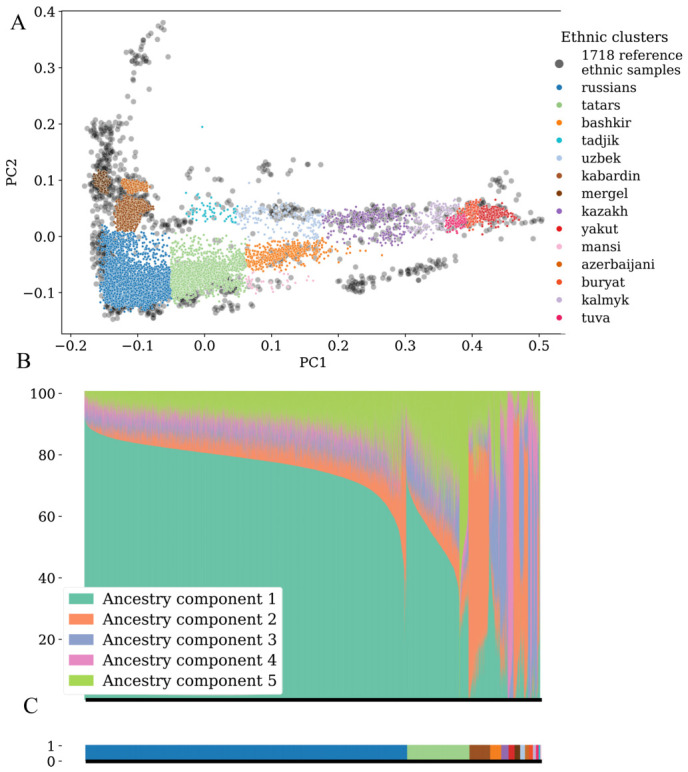
Population structure of the study cohort. (**A**) PCA projection of the cohort; colors indicate population clusters, with reference ethnic samples shown in gray. (**B**) ADMIXTURE analysis of ancestry components, where each bar represents an individual sample decomposed into ancestral component contributions. (**C**) Colors in the bar chart correspond to the population cluster legend in the PCA plot.

The study was carried out on an ethnically diverse cohort of 18,548 whole-genome sequencing (WGS) samples at 30× coverage. To characterize HLA distribution across different populations we started with characterisation of the cohort population structure. Genetic clusterisation (see Methods) produced 14 distinct ethnic clusters. (Table 1, Figure 1A).

To better characterise obtained clusters and to connect them with global populations, we performed the additional ADMIXTURE analysis. The analysis confirmed the internal homogeneity of identified groups with similar component distributions within individual clusters (Figure 1B). Cross-population ancestry decomposition revealed varying contributions of major continental ancestries in line with previously published data. Russians, the largest subgroup, was mostly composed of ancestry component 1, reflecting an apparent European ancestral component (mean proportion: 76.6%, SD: 7.8%), with an admixture of ancestry component 2 (mean: 8.5%, SD: 6.7%) that was predominant in Caucasus ethnic clusters (megrel, kabardin, azerbaijani). In contrast, megrel, kabardin, and azerbaijani groups exhibited a reverse admixture pattern, characterized by a high proportion of ancestry component 2 as the dominant component (67.0–87.9%), combined with a significant ancestry component 1 (European) admixture (6.7–16.2%). This component combination was consistent with expected admixture patterns in European-Caucasus populations [13]. The “Eastern” clusters: yakut, buryat, tuva were defined by ancestry components 3 and 4. The tatars group was mainly a mixture of ancestry components 1 (European) and 5.

These results are concordant with previous studies: five ancestral components were optimal, and the Russian cluster demonstrated a gradient from Northern European to Eastern patterns [14].

### 2.2. High Correlation of Allele Frequencies with Public Data Supports Ethnic Clusterisation and HLA Typing Approach

To ensure the reliability of our HLA-typing results and population stratification, we compared the observed HLA allele frequencies (AF) with those reported in publicly available sources (see Methods). Concordance primarily serves as supportive evidence of consistency with previously published data rather than as strict validation, due to the limited availability of public data.

**Figure 2:**
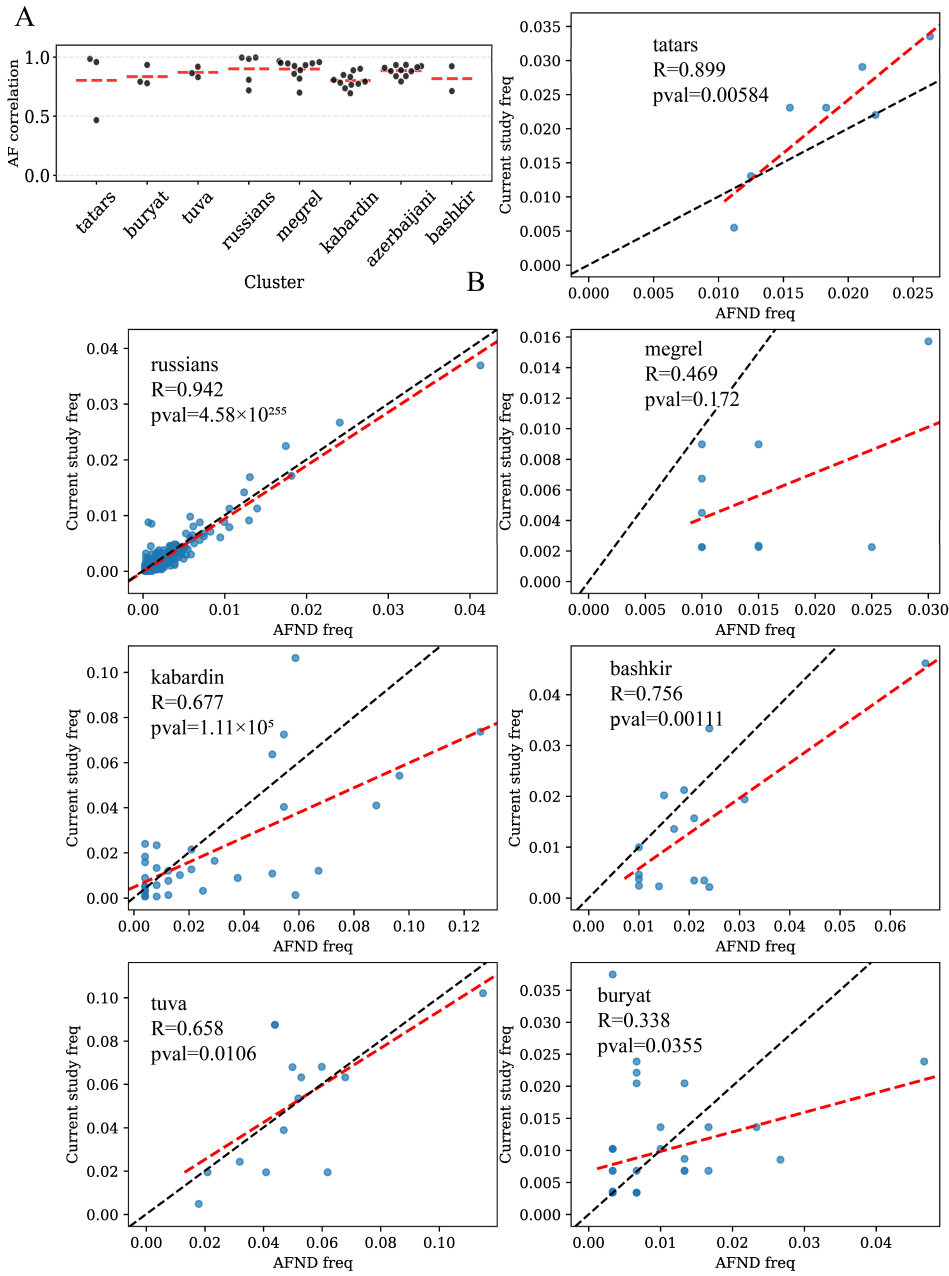
Concordance between the current study and AFND statistics. (**A**) Allele frequency correlation of the current study clusters and multiple AFND populations. Each dot represents a pairwise correlation of the current study genetic population cluster allele frequencies versus an AFND dataset. Mean allele-frequency correlation per genetic population cluster is shown by red dashed lines. (**B**) Frequency correlation of the current study cluster haplotypes and AFND population haplotypes, with the black dashed line representing perfect agreement (y=x) and the red dashed line showing linear approximation.

Only 8 out of 14 clusters had a complement dataset in the AFND with a sample size of at least 100 samples. As expected, the largest clusters, russians and tatars, showed the highest observed pairwise correlation coefficients (Figure 2A, Figures S4–S14). However, several AFND datasets exhibited systemic discordance in rare allele frequencies (AFND Russia Bashkortostan, Tatars; Russia Nizhny Novgorod, Russians; Russia Bashkortostan, Bashkirs; Figures S4, S7 and S13). Notably, each of these datasets was generated using NGS platforms, in contrast to the alternative HLA typing methods employed for other populations (Table S2). Despite observed heterogeneity in allele frequency correlations across study populations (SD: 0.04–0.29) all clusters maintained a mean Pearson correlation coefficient exceeding 0.8 (Table S3).

We then assessed the reliability of our HLA genotyping data by reconstructing haplotype frequencies and comparing them to reference data from AFND. Due to sparse haplotype records in AFND compared to allele data (Table S2), haplotype frequency distributions are less consistent across clusters, except for the russians group, which exhibits the highest correlation (R = 0.942, Figure 2B) likely due to both its homogeneous internal population structure and significantly larger sample size in the compared data. Smaller clusters tend to have fewer haplotype frequency records in AFND and also lack well-matched reference populations, thereby reducing the reliability of correlation estimates.

To summarize, when overlapping overlapping populations between our cohort and AFND we saw a high allele frequency correlation which supports the reliability of our data. However, haplotype frequencies show limited concordance. This issue particularly affects small clusters (Figure S15) and likely reflects the strong impact of sample size on statistical phasing accuracy.

### 2.3. Frequency Saturation Analysis Reveals Reduced HLA Diversity of the Yakut Population Cluster

**Figure 3:**
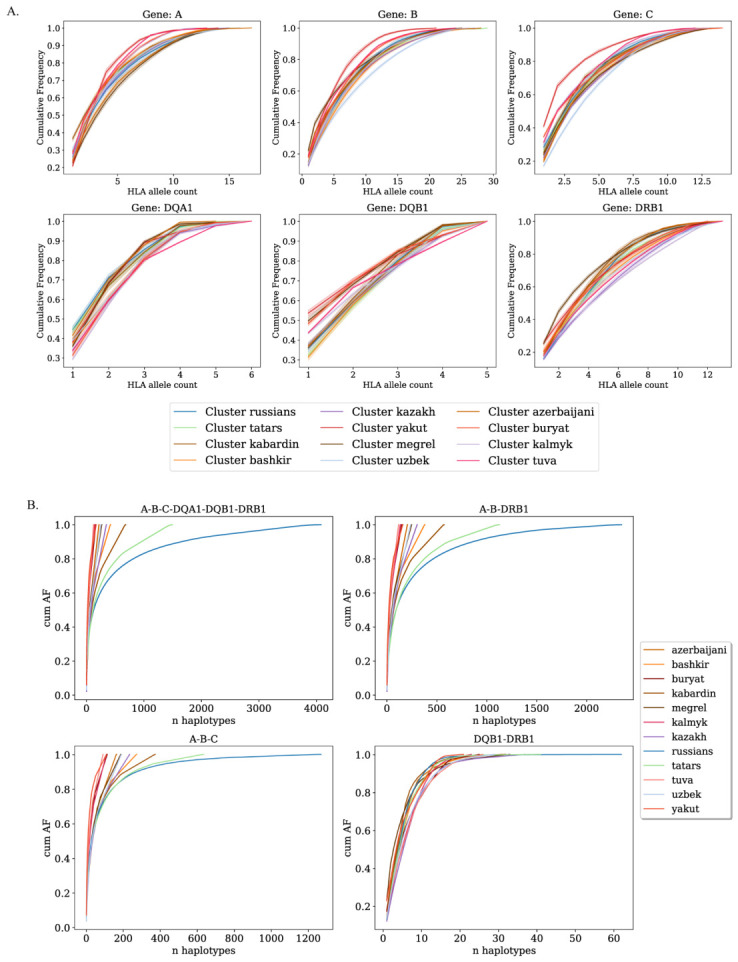
Cumulative HLA allele and haplotype frequencies. (**A**) Cumulative HLA population allele frequencies per HLA locus. Note the prominent red line representing the yakut genetic cluster for genes B and C. (**B**) Cumulative frequency plots for haplotypes of various genetically defined population clusters. A-B-C and DQB1-DRB1 haplotypes, though not clinically relevant, were considered for HLA class I and class II diversity comparison.

Because of the extreme variability of HLA alleles, large cohorts are required for diversity characterisation. Chao1 estimates of allelic richness (Table 2) show that the genetic diversity within the studied cohort is effectively represented across both the large and comparatively smaller identified clusters. We also analyzed the cumulative frequencies of alleles and haplotypes (Figure 3). Locus-wise curve matching revealed homogeneity in HLA allele cumulative frequency profiles across most clusters especially when it comes to HLA class II loci. However, the yakut genetic population cluster exhibited notable divergence in HLA-A, HLA-B and HLA-C allele frequencies (Figure 3A). The buryat cluster demonstrates a similar pattern to yakuts, consistent with its Asian origin.

Next we assessed whether clinically relevant haplotypes show cross-population variability. Additionally we included separate HLA class I and II haplotypes in analysis to estimate class contribution to population divergence (Figure 3B). Since the number of IMGT HLA [15] entries for class I is twice larger than for class II, HLA class I exhibits higher diversity than class II. Consequently, a frequency plateau is not detectable for class I-containing haplotypes (A-B-C, A-B-C-DQA1-DQB1-DRB1,A-B-DRB1) in most populations due to insufficient sampling (Figure 3B). This effect is roughly observable in the russians and tatars clusters only, where sample sizes were adequate to capture the asymptotic stabilization of the curve.

Thus, the allele frequency data can be considered representative. That allows us to infer a more modest allele diversity among yakut samples. Data on the frequencies of clinically relevant haplotypes remain insufficient for robust inter-population analysis.

### 2.4. Type One Diabetes-Associated HLA Haplotypes Differentially Represented Across Genetic Population Clusters and Shape Polygenic Risk Score Distribution

HLA alleles represent one of the strongest genetic determinants of immune regulation and disease susceptibility. Thus population HLA frequency differences should be reflected in the mean population risks of immune disorders. The substantial contribution of HLA loci to these risk estimates is particularly evident in type 1 diabetes mellitus (T1D).

**Figure 4:**
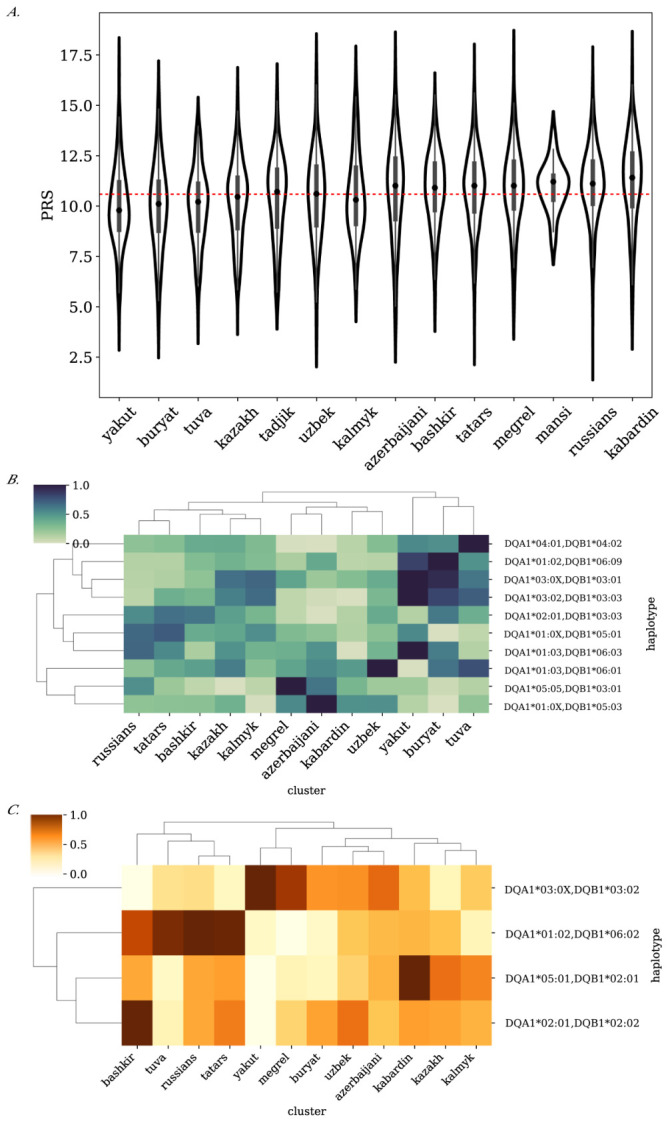
Distribution of T1D genetic risk score and associated HLA haplotypes across genetic population clusters. (**A**) PRS T1D distributions across population clusters. Dashed lines show mean values of T1D PRS across all populations. (**B**) Frequencies of T1D-protective HLA haplotypes (negative PRS weights), min-max scaled per haplotype across population clusters. (**C**) Frequencies of T1D-risk HLA haplotypes (positive PRS weights), min–max scaled per haplotype across population clusters.

Firstly, we calculated PRS for type 1 diabetes (T1D) PGS000024 [16] in our cohort. The score PGS000024 was chosen because it explicitly includes HLA alleles and their interaction instead of proxy SNPs. Previously inferred genetic ethnic groups demonstrated 29 statistically significant pairwise differences based on mean PRS. (Figure 4A, Table S4). The most explicit PRS distinction was observed between European and Caucasus ethnic groups when compared to East Asian groups. This comprised the tuva, yakut, and buryat clusters, which aligns with established patterns of HLA allele frequencies [15].

Secondly, we estimated the contribution of different HLA alleles into the PRS of different ethnic clusters. PGS000024 accounts for both risk and protective HLA alleles. Although a substantial proportion of the inferred haplotypes showed limited concordance with the AFND datasets, reflecting insufficient sample sizes in both the AFND and current study datasets for robust concordance assessment, class II HLA haplotypes demonstrated saturation across all population groups analyzed in the current study (Figure S16). Therefore, saturation may serve as an indirect indicator of representativeness for T1D-associated protective and risk Class II HLA haplotypes.

Protective HLA haplotype distribution across ethnic clusters demonstrated the same differences between large European and Asian groups as the overall PRS (Figure 4B). The “Asian” clusters (yakut, tuvan, buryat), which exhibit the lowest aggregate risk scores, form a distinct group defined by a high prevalence of established protective haplotypes. In contrast, T1D risk haplotypes demonstrate different distributions within large genetically defined ethnic groups (Figure 4C). While yakuts and buryats remain together, tuva cluster is closer to russians, bashkirs, and tatars. Kabardin group, characterized by the highest mean PRS, demonstrates a highly expressed risk haplotype DQA1*05:01~DQB1*02:01, which separates this group from other lower-risk clusters. Overall differences in the HLA allele frequencies occasionally result in a similar PRS score which is based on a different set of risk and protective alleles. For example, megrel and tatars, while exhibiting statistically indistinguishable PRS, have completely different HLA profiles. Within the protective haplotype set, the megrel cluster is characterized by high-frequency haplotypes DQA1*05:05~DQB1*03:01 and DQA1*01:0X~DQB1*05:03. In contrast, the tatar profile is dominated by DQA1*01:0X~DQB1*05:01 and DQA1*02:01~DQB1*03:03. In the case of risk haplotypes, the megrel group shows a pronounced prevalence of DQA1*03:0X~DQB1*03:02, which brings this cluster closer to yakut profiles than to tatars (DQA1*01:02~DQB1*06:02 is predominant).

These results indicate that identical aggregate risk estimates can mask a potentially different immunogenetic background specific to the population.

### 2.5. Discovering New HLA Alleles in Mansi Cluster

Small, genetically isolated ethnic groups remain underrepresented in genomic studies, yet they hold significant potential for uncovering novel allelic variants. Leveraging the advantage of a large cohort, we tried to estimate the prevalence of a new allele in a poorly described population. The Mansi are a small (around 12,000) indigenous Ugric ethnic group living in Western Siberia, Russia, primarily in the Khanty-Mansi Autonomous Okrug–Yugra [17]. We applied T1K and identified 232 potentially novel SNPs that passed default filters in a mansi group in our cohort (38 individuals). While the majority of these were variants in pseudogenes, there were 2 previously unreported genetic variants across the target HLA class I and II loci (A, B, C, DQA1, DQB1, DRB1), carried by 2 individuals (Table 3).

Although further validation is required, these findings highlight the presence of population-specific HLA alleles that may reflect unique selective pressures or founder effects within this cluster and gives an opportunity for further expanding the IMGT HLA database.

## 3. Discussion

The present study has provided an overview of HLA allele and haplotype frequencies across different Russian population groups, contributing to the understanding of genetic diversity, population structure, and future disease susceptibility, transplantation medicine, or anthropological studies.

Given the substantial sample size and uniform HLA typing method, our dataset provides broader coverage of Russia’s major populations, including smaller ethnic groups than previously published research, which focuses on individual populations or does not subdivide large multinational cohorts [18,19,20,21]. However, the cohort was derived from routine commercial sequencing and was not designed as a fully comprehensive demographic survey. Consequently, potential recruitment biases related to geographic distribution, socioeconomic factors, and uneven representation of regional demographic features cannot be excluded, which affect the generalizability of the observed allele and haplotype frequencies.

Ethnic groups were identified exclusively by genetic clusterisation. This methodology is a scalable framework for participant recruitment, which allows researchers to conduct studies prior to resource-consuming ethnographic expeditions. Genetic clusterisation also provides measurable, objective information compared to the self-reporting ethnicity. At the same time, since sample recruitment did not account for genealogy or geographic origin, the resulting groups exhibit admixture of varying origins, potentially blurring the borders of distinct population clusters. Moreover, cluster size imbalance between subpopulations may affect representativeness of populations. Therefore, the identified clusters should be interpreted primarily as genetically inferred groups within the current cohort rather than as fully representative descriptions of the corresponding populations in Russia. Nevertheless, the derived clusters encompass both European-related groups (e.g., russians, kabardin) and Asian-related populations (e.g., yakut, tuva), reflecting the country’s diverse genetic landscape.

Frequency data presented here align well with allele frequency from AFND. Nevertheless, AFND exhibits several notable limitations including modest sample size for some populations and lack of the other populations. Due to these constraints only 8 ethnic clusters out of 14 identified have corresponding AFND entries to compare. Despite good overall correlation, some NGS-derived datasets from the AFND have demonstrated notable discordance (median R = 0.71) in reported allele frequencies, which may be due to insufficient quality control of the submitted datasets. A clear data inconsistency is evident in the AFND Russia Bashkortostan, Tatars dataset. The reported frequency for the allele DQB1*03 is only 0.0026. This is highly questionable, as the frequency of the DQB1*03:01 allele in the same AFND dataset is 0.1875. Since the general allele group DQB1*03 must include all its subgroups (e.g., DQB1*03:01, DQB1*03:02), its frequency cannot be lower. Several genetic population clusters (e.g., azerbaijani and kabardin clusters) lack specific corresponding representative AFND reference data. The DQA1 data for the tuva and buryat clusters are constrained to a single available AFND dataset without independent validation from additional up-to-date reference HLA typing data. Such pronounced inconsistencies and data sparsity clearly show the need for a larger, uniform data source.

We also analysed haplotype concordance. Haplotypes offer greater informational density than single alleles for enhanced diagnostics and population analysis [22,23]. However, technological challenges in haplotype phasing have limited the availability of haplotype data compared to genotype data. Insufficient data reliability stems from key constraints: sparser haplotype representation in AFND than allele data, ambiguous AFND inference methodologies, scarce data for Russian ethnic minorities, and phasing biases in EM-based refinement strategies. Hence, meaningful comparative analysis is only relatable for the largest groups in our datasets-russians and tatars. In the others, comparisons reveal only relative concordance. Despite substantially greater sample recruitment compared to previous AFND entries and the ability to capture HLA allele frequency diversity, this study does not capture the full HLA haplotype repertoire of minor ethnic clusters.

Since our data demonstrate a sufficient level of concordance with previous studies while also benefiting from a uniformly typed large ethnic sample and a high-resolution method for processing genetic data, we tried to assess the diversity of alleles and haplotypes in different genetically defined ethnic groups and estimate inter-population variability. Analysis showed that the yakut cluster exhibits a lower HLA allele diversity relative to other studied clusters. Seemingly, HLA haplotypes and alleles show evidence of founder effect followed by genetic drift which is supported by earlier studies of Y-/MT-haplogroups [24,25] and can be an important feature for optimal donor-recipient matching.

The identification of previously undescribed HLA alleles is even more critical for optimal donor-recipient selection. A subset of samples from the current study cohort, belonging to the russians genetic population cluster, was utilized in a study focused on the discovery of novel HLA alleles using 30× WGS data. The identified variants were validated by Sanger sequencing and submitted to the IPD-IMGT/HLA Database [26]. However, novel allele discovery is particularly relevant for minority ethnic groups in Russia. Our study provides an example of a search for potentially novel alleles within a distinct narrow subpopulation of the current cohort. The mansi group (38 samples) exhibited two potentially novel alleles (0.44% of all alleles considered). This observation cannot be readily extrapolated to the general Mansi population and should therefore be regarded as a preliminary finding requiring further Sanger-based validation. Nevertheless, it is an example highlighting the underrepresentation of minor ethnic groups in public datasets.

Population characteristics of HLA allele distribution can bias PRS. Statistically significant differences across population clusters were observed in T1D PRS (PGS000024), largely driven by the HLA component. The tuva, buryat, and yakut clusters showed lower average risk than the russians cluster, aligning with regional epidemiology [27]. However, the kabardin cluster’s PRS deviated from expected risk, despite typical regional T1D incidence [27]. The observed results could stem from both sample composition (genetic vs. geographic clustering) and the acute need of population-dependent distinct score corrections. Importantly, even statistically similar risk scores can result from different underlying HLA haplotype distributions, suggesting possible population-specific etiopathogenetic mechanisms. Thus, describing ethnic variability in HLA haplotypes and implementing population stratification are essential for appropriate PRS implementation. However, these findings should be interpreted cautiously. The present PRS analysis is primarily illustrative and is intended to demonstrate population-specific differences in risk variant and HLA allele distributions rather than provide clinically applicable risk prediction.

Thus we’ve created a novel, uniformly processed genomic dataset of HLA allele and haplotype frequencies encompassing a wide spectrum of Russian ethnicities, which has been submitted to AFND. The current study contributes to public genomic databases that have notable deficiency of representative samples and can serve as a potential reference for further population genetics and epidemiology studies.

## 4. Materials and Methods

### 4.1. Cohort Description

Peripheral venous blood samples were collected from 18,548 participants (44.6% male, 55.4% female) by LCC “Evogen” during routine sequencing conducted between 4 April 2025 and 1 December 2025. All participants were healthy individuals from the Russian population and provided written informed consent.

### 4.2. Sample Preparation and Sequencing

DNA extraction was performed by spin column using the Qiagen QIAamp DNA Blood Kit (Cat. No. 51106) (Qiagen, Hilden, Germany) from whole blood according to the manufacturer’s protocol. DNA amount was measured fluorometrically with Qubit4 (Thermo Fisher Scientific, Waltham, MA, USA)/Denovix (DeNovix Inc., Wilmington, DE, USA). For subsequent library preparation, only genomic DNA of high quality ($OD_{260}/OD_{280}=1.8$–2.0, $OD_{260}/OD_{230}>2.0$) was used. Library preparation was performed with a PCR-free enzyme fragmentation protocol (MGIEasy FS PCR-Free DNA Library Prep Set, Cat. No. 1000013455) using 800–1200 ng gDNA. The distribution of insert size was 400–600 bp. WGS library preparation was performed both manually and automatically.

Whole genome sequencing was performed using DNBSEQ-G400 (MGI Tech Co., Ltd., Shenzhen, China) with FCL PE150 (cat. no. 1000012555), FCL PE200 (cat. no. 1000013858), and DNBSEQ-T7, according to the manufacturer’s protocol.

### 4.3. WGS Processing

Raw FASTQ files were processed using a modified version of the Broad Institute’s Whole Genome Germline Single Sample pipeline [28] for quality control, mapping, and variant calling. A key modification involved replacing BWA-MEM2 with Minimap2 [29] to optimize alignment. Subsequent analyses included population structure estimates with principal component analysis (PCA) and ADMIXTURE v.1.3.0 [30] in unsupervised mode.

### 4.4. HLA Calling and Haplotype Inference

NGS-based HLA typing was performed for all samples using HLA-HD (IMGT release 3.53.0) [31] with the default parameters (-m 100 and -c 1.0). Although HLA-HD does not explicitly report locus-level coverage statistics in its output, the tool internally utilizes Bowtie 2 to remap reads originating from the HLA region against the IMGT reference database. We have used the same mapping strategy and alignment stage as HLA-HD to estimate average locus coverage across the test cohort [26]. The distribution of mean coverage depths across loci was centered around 15–17× (Figure S1).

T1K [32] was also applied (using –preset hla-wgs parameter) to assess concordance with HLA-HD typing in a random subset of 100 samples from the cohort. The comparison demonstrated an overall concordance rate of 97%. The distribution of T1K HLA typing scores is shown in Supplementary Figure S2, the typing rate (quality score > 0) was 96%.

Since T1K supports detection of novel HLA variants and SNPs, it was further applied to one cluster presumed to represent a genetically isolated population group.

Haplotype inference was performed using Hapl-o-Mat v1.2.2 [33].

### 4.5. Ethnic Annotation and Clustering

We performed genetics-based population labeling using a reference panel of 1718 samples from previously published Russian population genetic studies (Table S1) [24,34,35,36,37]. 38,316 SNP that were shared across different technologies in these datasets were retained. Reference samples were clustered in two steps: firstly, DBSCAN (scikit-learn DBSCAN; eps = 0.11) on the first two principal components identified 40 clusters; secondly, agglomerative clustering (scikit-learn AgglomerativeClustering; distance_threshold = 0.1) of cluster centroids refined these to 19 final clusters (Figure S3). Thresholds were optimized using silhouette scoring.

The resulting reference clusters (paper-based) were used to fit a Random Forest classifier (RandomForestClassifier module, scikit-learn) and assign 18,548 individuals from the target dataset to these paper-based groupings. Cluster labels were assigned according to the predominant population representatives within each cluster and should therefore be interpreted as genetic affinity labels rather than definitive ethnic or self-identified population categories. Because of the lack of self-reported ethnicity, detailed genealogical information, or fine-scale geographic metadata were available for the target cohort, the inferred clusters reflect patterns of genetic similarity only and may not fully correspond to socially, culturally, or historically defined ethnic groups. To distinguish inferred population clusters from the general ethnic groups discussed in the text, cluster designations are presented in lowercase, whereas ethnic group names are capitalized.

### 4.6. ADMIXTURE Decomposition

Genetic ancestry and population structure were further characterized using ADMIXTURE v1.3.0 [30], employing the same variant set utilized for PCA. The optimal value for K (ranging from K = 2 to K = 10) was determined by minimizing the five-fold cross-validation error.

### 4.7. HLA Haplotype Inference

Haplotypes were inferred using Hapl-o-mat [33]. The analyzed haplotype blocks, primarily HLA-A-B-DRB1, and extended versions such as HLA-A-B-C-DRB1 or HLA-A-B-C--DRB1-DQ- were defined by clinical guidelines and published evidence [38,39,40,41].

### 4.8. External Cohorts and Correlation Analysis

External cohorts were obtained from AFND [5] and are described in Table S2. We used these datasets to estimate concordance with previously published HLA frequencies. To maximize the utilization of heterogeneous data from the AFND, we performed correlation analyses across all available resolutions and typing methods. This approach was chosen to prevent the significant data loss as a result of restricting the analysis to a single resolution or protocol. We also restricted our analysis to datasets with a sample size greater than or equal to 100. For the comparison of haplotype frequencies we selected AFND entries that exhibited the highest allele frequency concordance with the current study clusters and for which haplotype data were available (N haplotypes > 5).

Pairwise correlation coefficients were calculated between the study-derived population cluster and each corresponding reference population available in the AFND. *p*-values were adjusted using the Bonferroni correction.

### 4.9. Analysis of Allele Richness and Allele/Haplotype Frequency Distributions

The proportion of distinct alleles detected was estimated as the ratio of observed allele number to the Chao1 estimator [42] per locus, which infers unseen alleles from the frequency of singletons and doubletons.

Cumulative frequency plots were constructed for each locus to estimate genetic diversity. Alleles/haplotypes were ranked by descending frequency and summed. The resulting sigmoidal curve shows the distribution: a steep rise indicates dominance by a few common alleles, while a gradual slope reflects more even frequencies. By overlaying these curves, we visually assessed similarity between populations.

To balance sample sizes, each cluster was subsampled 10 times to match the smallest group. For cumulative haplotype frequencies, however, the entire sample set was used, as sample size critically impacts Hapl-o-Mat estimates and haplotypes are generally more diverse compared to alleles.

### 4.10. Polygenic Risk Scores (PRS) Calculation and Ethnic HLA Associations

Individual PRS were computed as the sum of allele dosages weighted by effect sizes (β) from PGS Catalog [16] summary statistics. Pairwise Mann-Whitney U tests were used to compare the distribution of the Type 1 Diabetes PRS across study populations. *p*-values were adjusted using the Bonferroni correction.

Using PRS summary weights, T1D haplotypes were designated as risk (positive weight) or protective (negative weight). Their population-specific frequencies were min-max scaled per haplotype, then visualized and hierarchically clustered via seaborn.clustermap with default parameters.

## Figures and Tables

**Table 1:** Population cluster sizes.

Cluster	Size
russians	13,147
tatars	2512
kabardin	834
bashkir	442
kazakh	299
yakut	235
megrel	230
uzbek	198
azerbaijani	170
buryat	149
kalmyk	120
tuva	112
tadjik	62
mansi	38
Total	18,548

**Table 2:** Chao1 estimates of allelic richness in the study cohort.

Cluster	Mean	SD
azerbaijani	0.913	0.06
bashkir	0.965	0.033
buryat	0.934	0.066
kabardin	0.973	0.017
kalmyk	0.943	0.057
kazakh	0.962	0.042
megrel	0.962	0.033
russians	0.994	0.004
tatars	0.987	0.008
tuva	0.976	0.019
uzbek	0.937	0.051
yakut	0.972	0.035

**Table 3:** Exonic genetic variants in the mansi population cluster. The REF column refers to a reference allele from the IMGT HLA allele database. POS column shows coordinate in concatenated exon reference sequence.

Allele	POS	REF	ALT	n Reads Support Alt	n of Reads Cover This Position	n of Reads That Uniquely Aligned Support Ralt	The Coordinate with Respect to the Input Reference
HLA-B*35:500	617	G	C	10	12	3	1040
HLA-C*03:524	109	C	T	7	9	3	288

## Data Availability

Allele and haplotype frequency data supporting the findings of this study have been submitted to the AFND [5] and are available under accession IDs: 3848, 3849, 3843, 3841, 3845, 3851, 3847, 3852, 3840, 3842, 3844, and 3850. Individual-level genetic data are governed by a data use agreement and are not publicly available due to privacy and confidentiality restrictions. All Supplementary Tables and Figures associated with this paper are provided in the Supplementary Information files.

## Supplementary Materials

The following supporting information can be downloaded at: https://www.mdpi.com/article/10.3390/ijms27115063/s1.

## Abbreviations

The following abbreviations are used in this manuscript:

HLA	Human leukocyte antigen
AFND	Allele Frequency Net Database
WGS	Whole Genome Sequencing
PRS	Polygenic Risk Score
PCA	Principal Component Analysis
T1D	Type 1 Diabetes
