# Supplementary material for: HLA Alleles and Haplotype Distribution Across Russian Population Groups
Source: Int J Mol Sci. 2026 Jun 3;27(11):5063. doi: 10.3390/ijms27115063 (PMC13256726; doi:10.3390/ijms27115063)
Supplement: Supplementary file 1 [file ijms-27-05063-s001.zip › Supplementary_Figures_and_Tables.pdf]

HLA alleles and haplotype distribution across Russian population groups
Supplementary

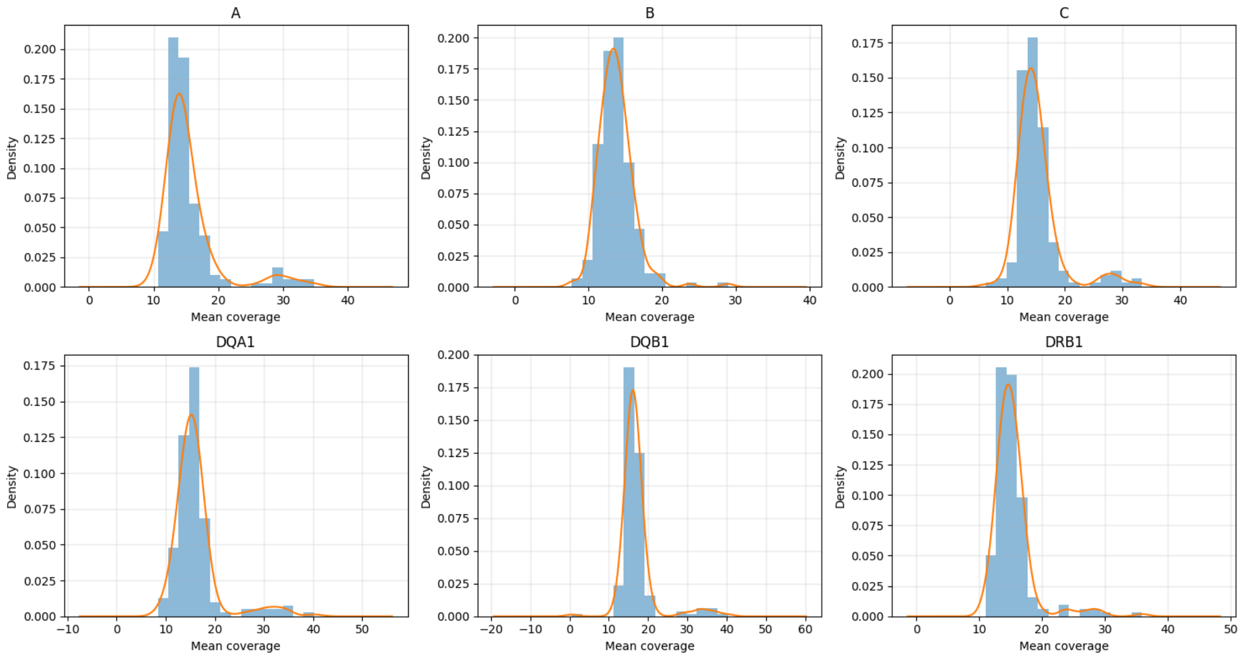

Fig. S1 Mean coverage depth distribution in HLA loci for a test sample of 100 random samples.

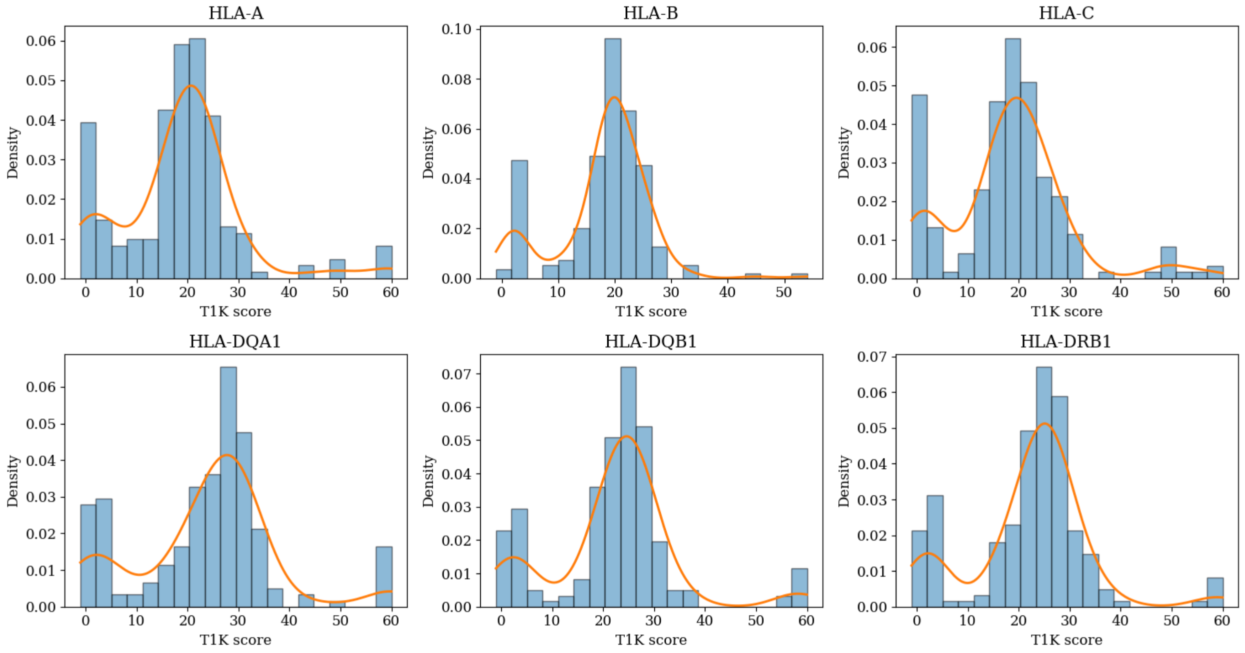

Fig. S2 T1K typing quality score distribution in HLA loci for a test sample of 100 random samples.

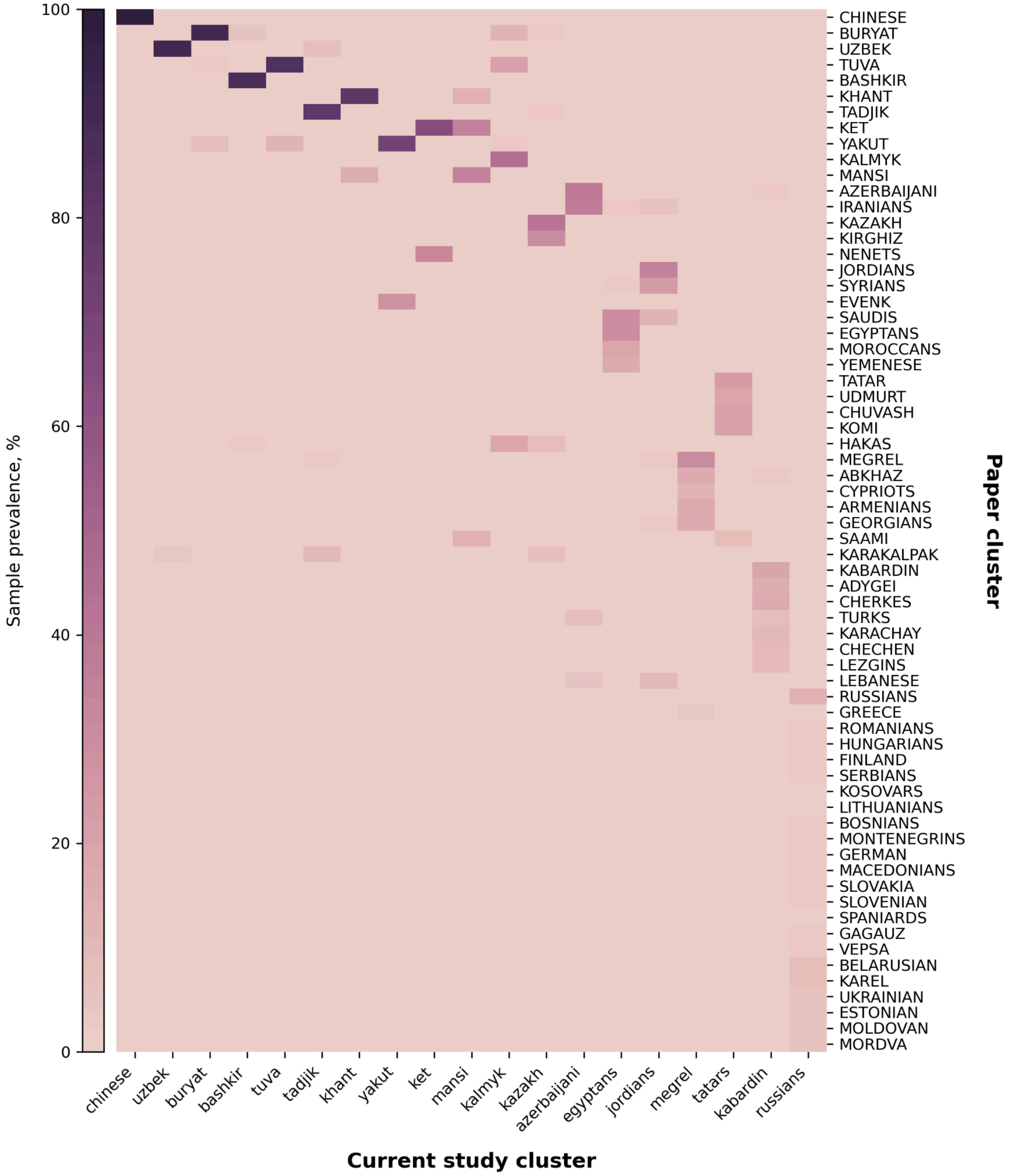

Fig. S3 Distribution of 1718 ethnically-annotated reference samples across current study genetic clusters. Columns represent the genetic clusters identified in the current study. Rows represent the ethnicity labels reported in the source publications. Each cell shows the percentage of samples within a given cluster (column) that were originally annotated with a specific ethnicity (row).

Table.S2. Population clusters of the current study and the corresponding populations from the AFND database. AFND HLA typing methods are NGS (Next Generation Sequencing), SBT (Sequence Based Typing), SSP (Sequence-Specific Primers), SSOP (Sequence-Specific Oligonucleotide Probes). Column AF/Haplotypes denotes if data for HLA alleles or haplotypes frequency is available. The Current Cluster column maps corresponding genetic groupings for direct frequency comparisons. The Resolution column specifies the degree of allelic typing quality in the AFND entry, expressed as the number of digits in the reported HLA genotype resolution.

AFND population	AFND sample size	AFND HLA typing method	Source	AF/ Haplotypes	Current Study Cluster	Resolution
Russia Bashkortostan, Tatars	192	NGS	Bone Marrow Registry	both	tatars	mixed(2-8)
Russia Tatars	355	SBT	Bone Marrow Registry	both	tatars	2d
Russia South Ural Tatar	135	SSP	Anthropology Study	both	tatars	mixed(2-4)
Russia Tuva pop 2	169	SSOP	Anthropology Study	both	tuva	mixed(2-6)
Russia Transbaikal Territory Buryats	150	SSOP	Bone Marrow Registry	both	buryat	mixed(2-4)
Russia Northwest pop 2	346	SSP	Controls for Disease Study	AF	russians	2d
Russia Moscow Pop 2	2000	SSP, SSOP	Bone Marrow Registry	both	russians	2d
Russia Moscow	2650	SSP, SSOP	Moscow Stem Cell Bank	AF	russians	2d
Russia Nizhny Novgorod, Russians	1510	NGS	Bone Marrow Registry	both	russians	mixed(4-8)
Armenia combined Regions	100	SSP	Bone Marrow Registry	both	megrel	mixed(2-4)
Georgia Tbilisi	109	SSOP	Anthropology Study	AF	megrel	mixed(2-8)
Russia North Ossetian	127	SSP, SSOP	Bone Marrow Registry	both	megrel	mixed(2-4)
Armenia combined Regions	100	SSP	Bone Marrow Registry	both	kabardin	mixed(2-4)
Georgia Tbilisi	109	SSOP	Anthropology Study	AF	kabardin	mixed(2-8)
Russia Bashkortostan,	120	NGS	Bone Marrow	both	bashkir	mixed(2-8)

Bashkirs			Registry			
Russia South Ural Bashkir	146	SSP	Anthropology Study	both	bashkir	mixed(2-4)
Russia North Ossetian	127	SSP, SSOP	Bone Marrow Registry	both	azerbaijani	mixed(2-4)

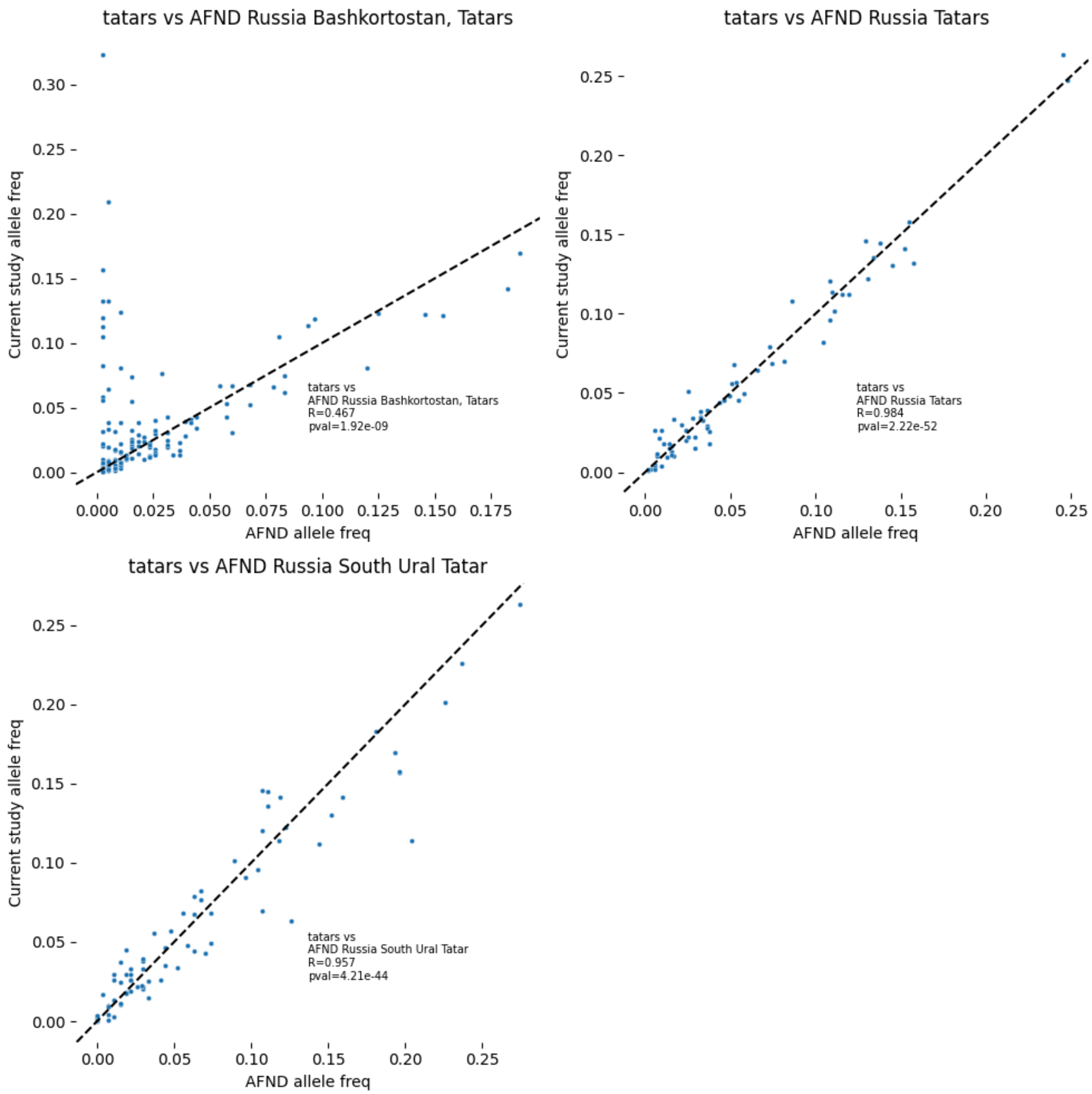

Fig S4. Allele frequency correlation of the current study cluster tatars and the corresponding AFND datasets, with the black dashed line representing the line of perfect haplotype frequency agreement (y=x).

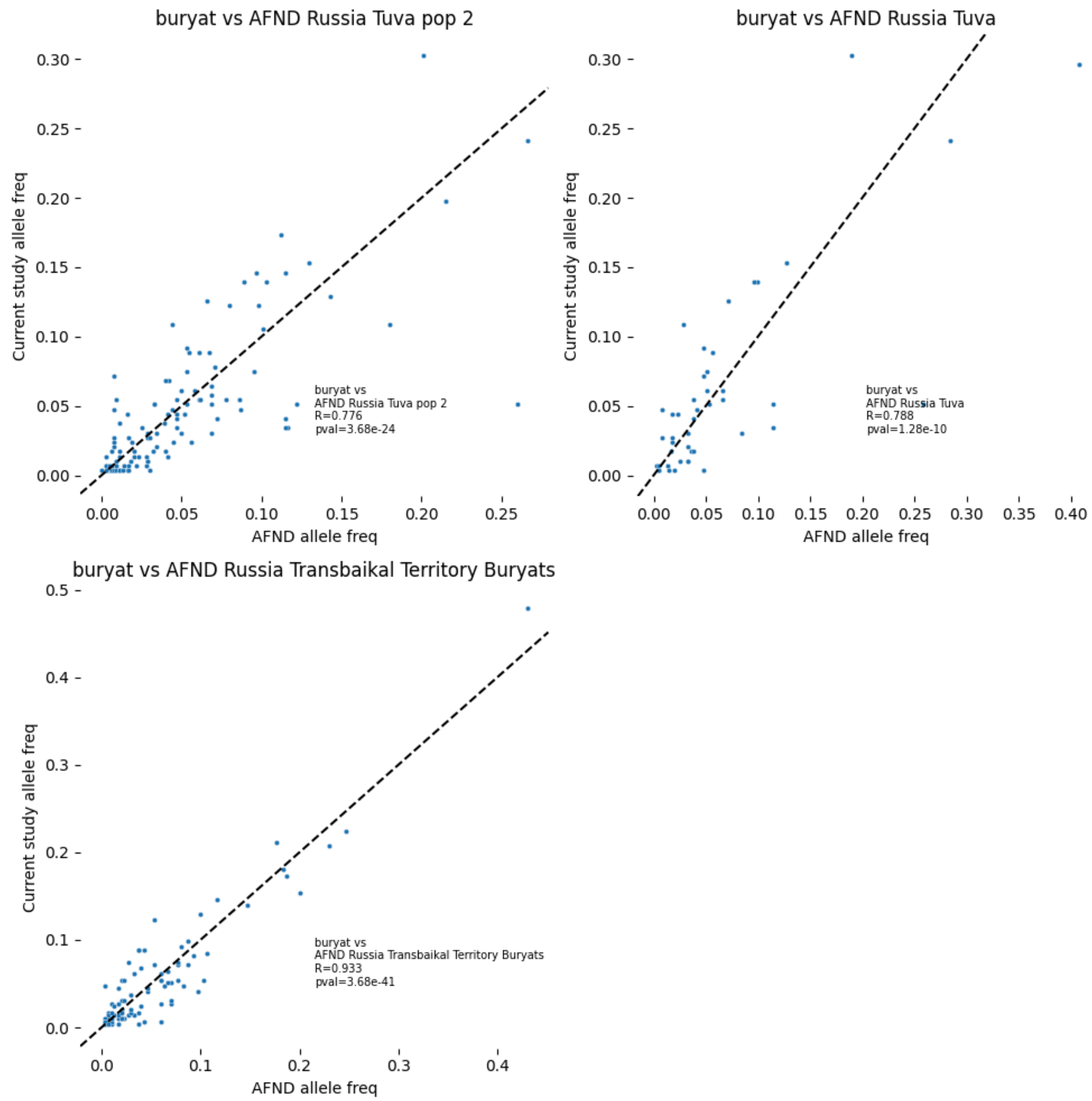

Fig S5. Allele frequency correlation of the current study cluster buryat and the corresponding AFND datasets, with the black dashed line representing the line of perfect haplotype frequency agreement (y=x).

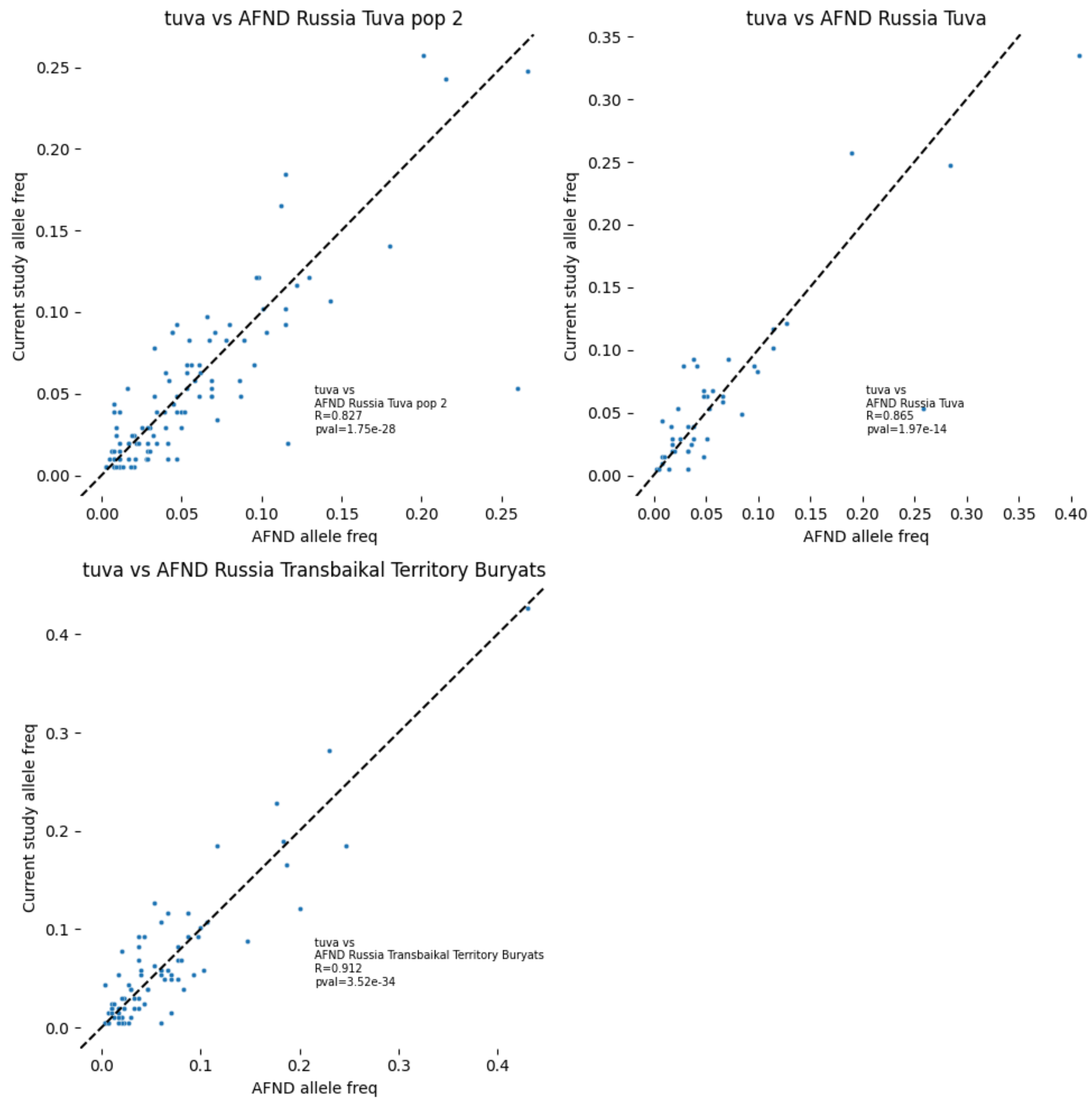

Fig S6. Allele frequency correlation of the current study cluster tuva and the corresponding AFND datasets, with the black dashed line representing the line of perfect haplotype frequency agreement (y=x).

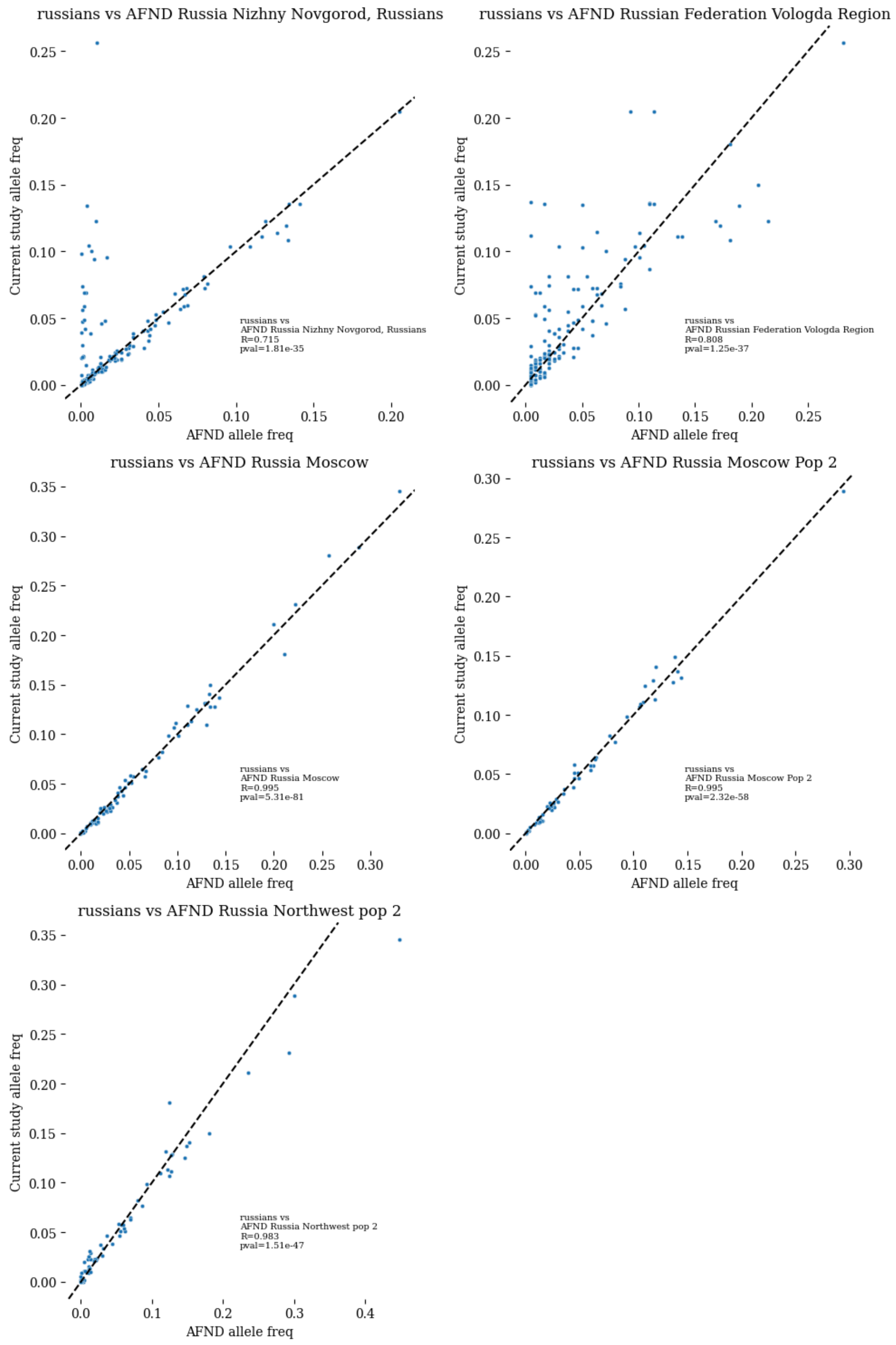

Fig S7. Allele frequency correlation of the current study cluster russians and the corresponding AFND datasets, with the black dashed line representing the line of perfect haplotype frequency agreement (y=x).

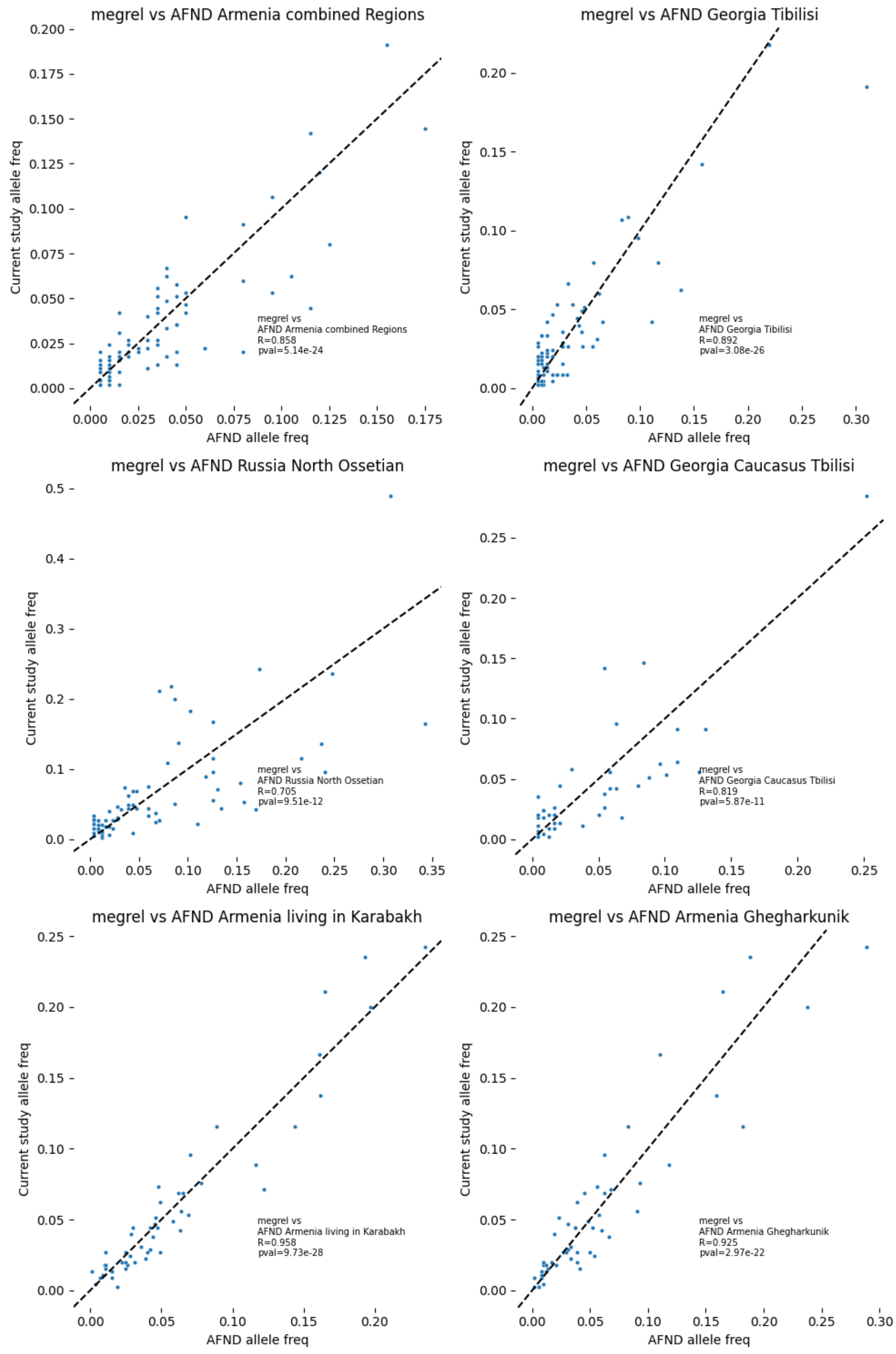

Fig S8. Allele frequency correlation of the current study cluster megrel and the corresponding AFND datasets, with the black dashed line representing the line of perfect haplotype frequency agreement (y=x).

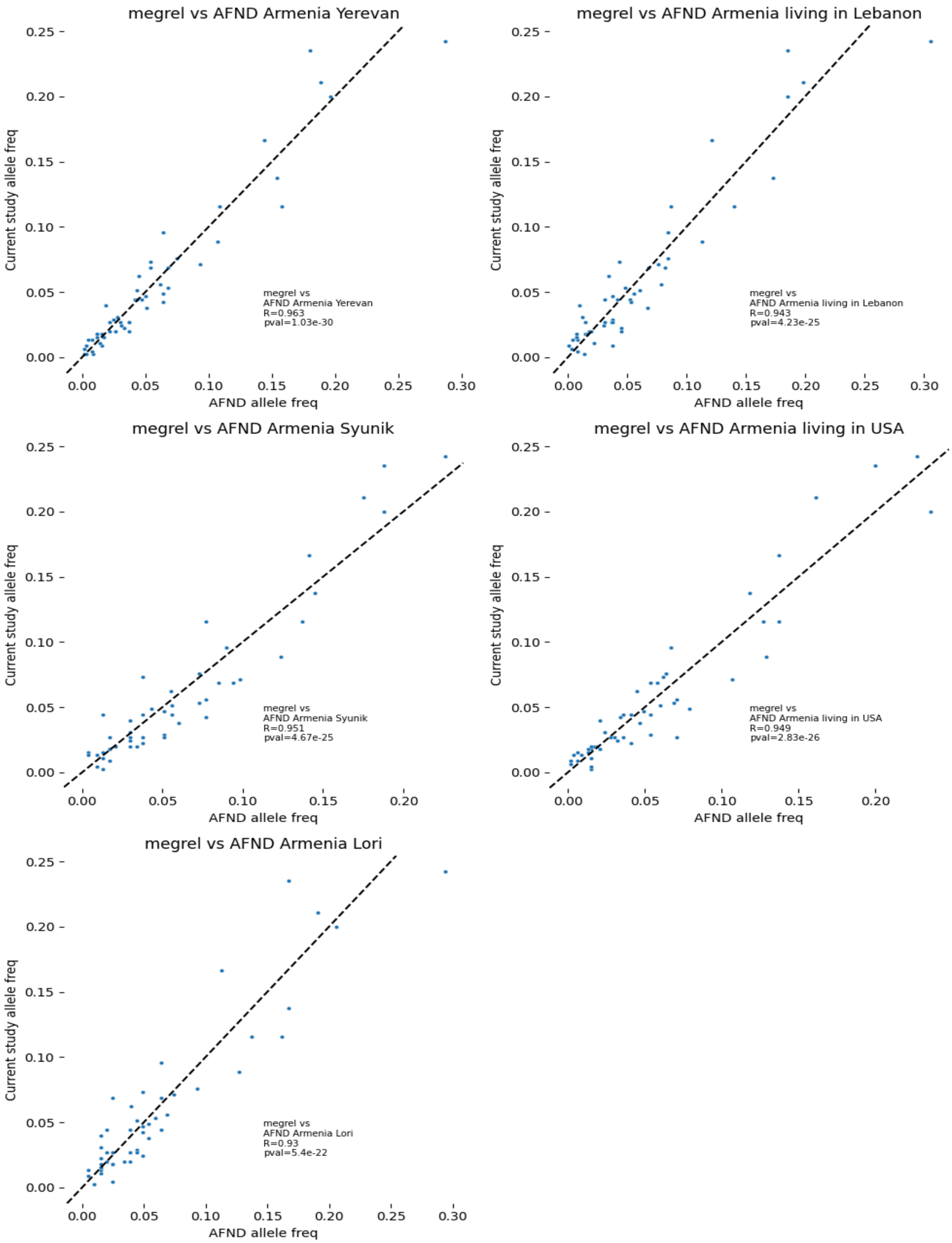

Fig S9. Allele frequency correlation of the current study cluster megrel and the corresponding AFND datasets, with the black dashed line representing the line of perfect haplotype frequency agreement (y=x).

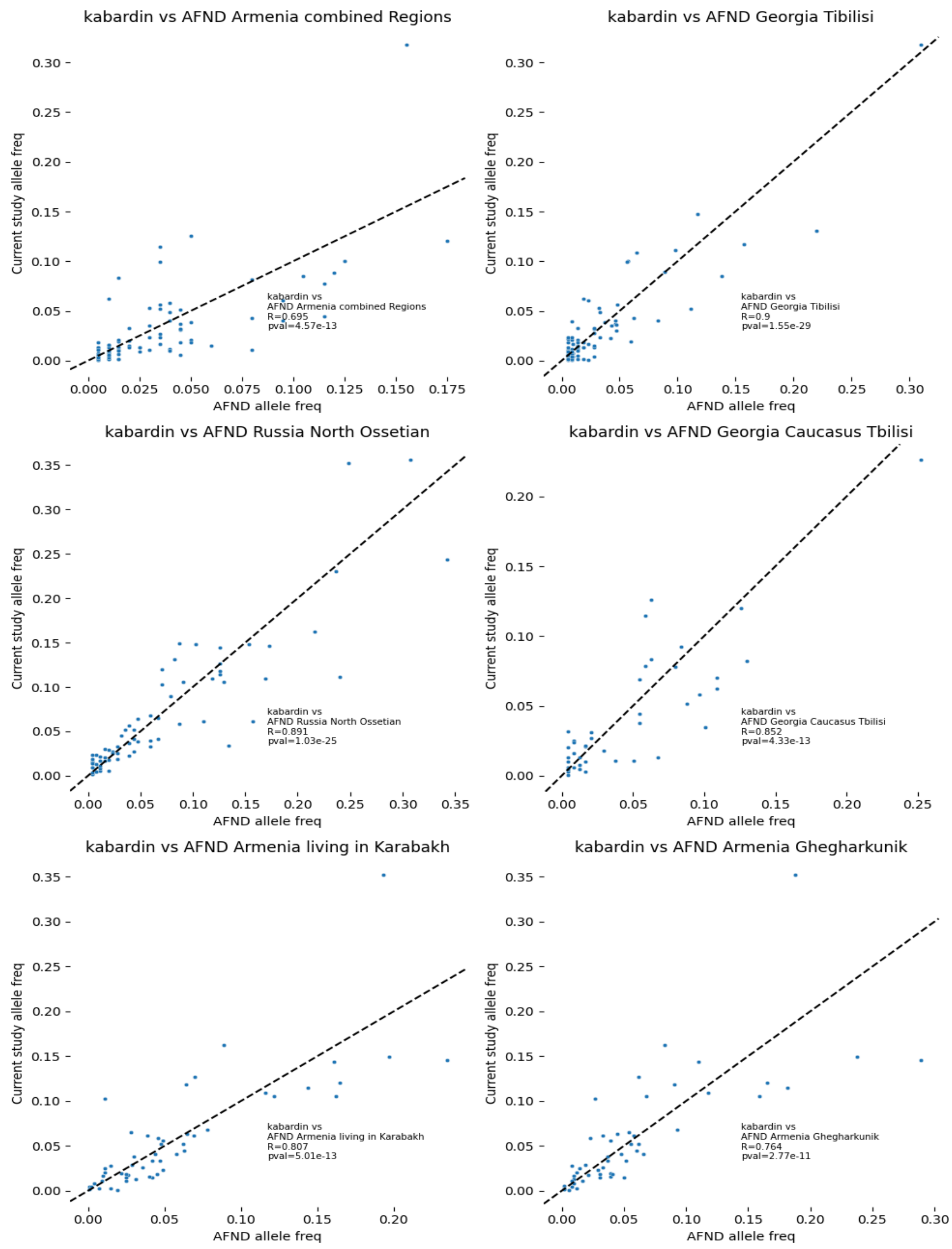

Fig S10. Allele frequency correlation of the current study cluster kabardin and the corresponding AFND datasets, with the black dashed line representing the line of perfect haplotype frequency agreement (y=x).

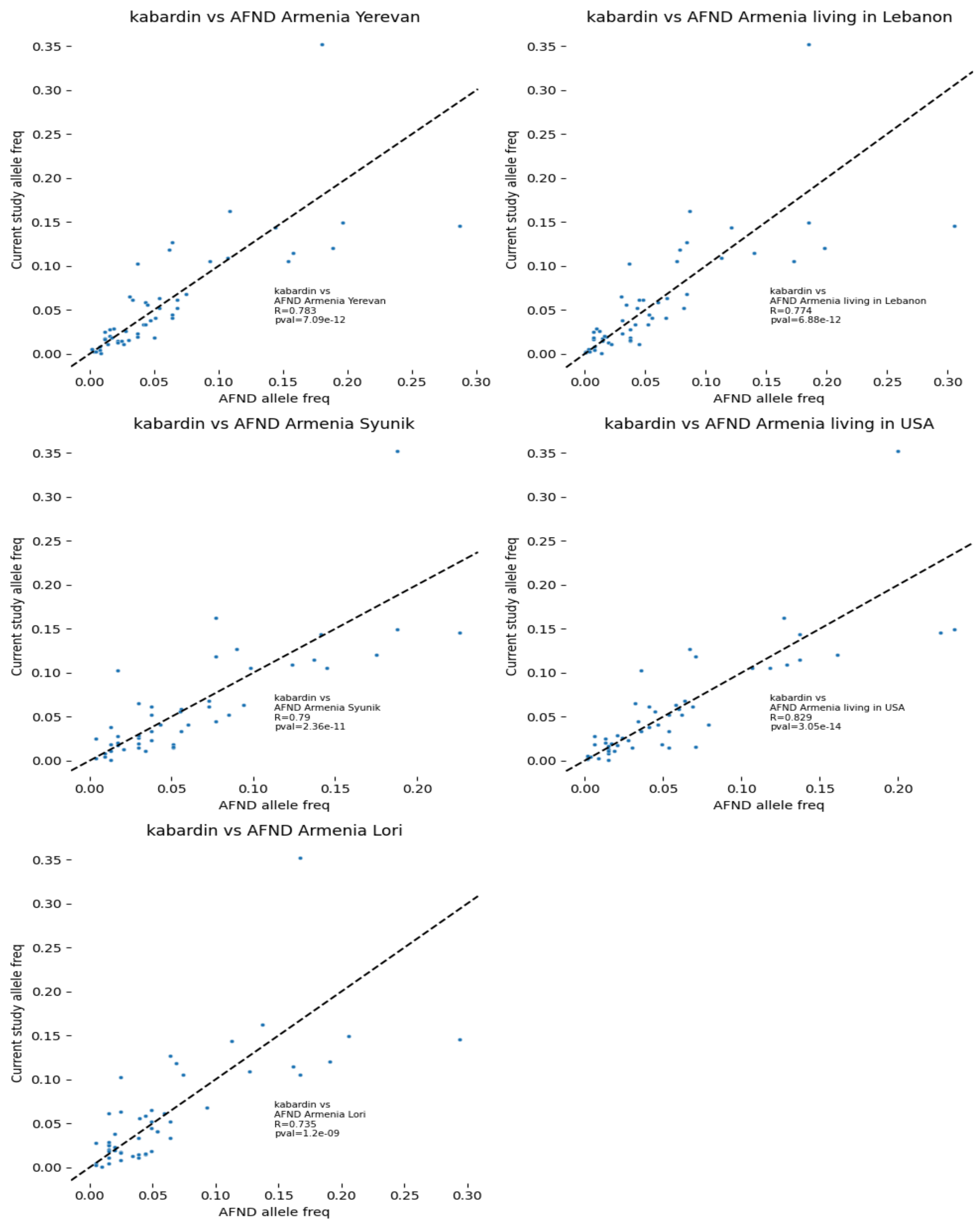

Fig S11. Allele frequency correlation of the current study cluster kabardin and the corresponding AFND datasets, with the black dashed line representing the line of perfect haplotype frequency agreement (y=x).

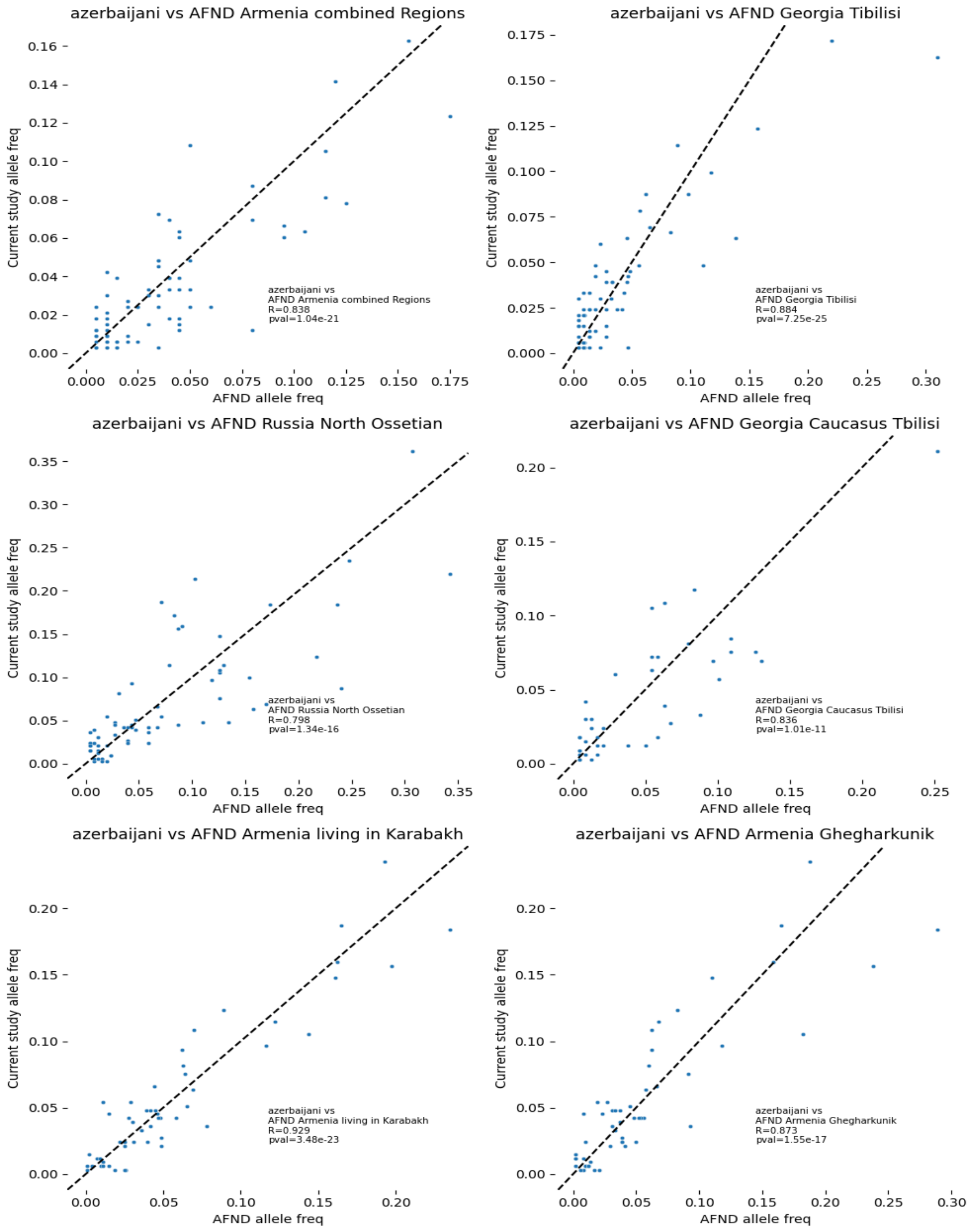

Fig S12. Allele frequency correlation of the current study cluster azerbaijani and the corresponding AFND datasets, with the black dashed line representing the line of perfect haplotype frequency agreement (y=x).

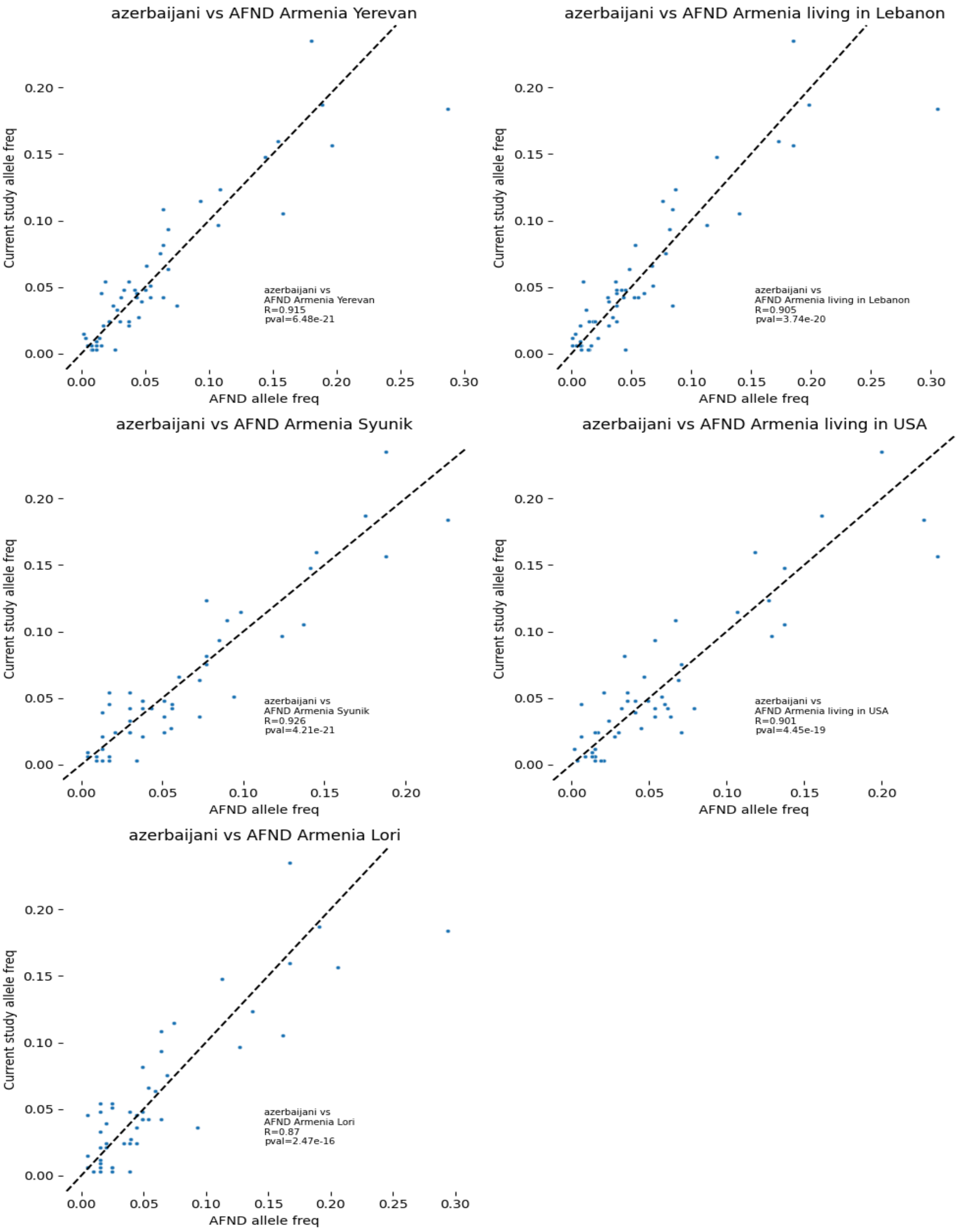

Fig S13. Allele frequency correlation of the current study cluster azerbaijani and the corresponding AFND datasets, with the black dashed line representing the line of perfect haplotype frequency agreement (y=x).

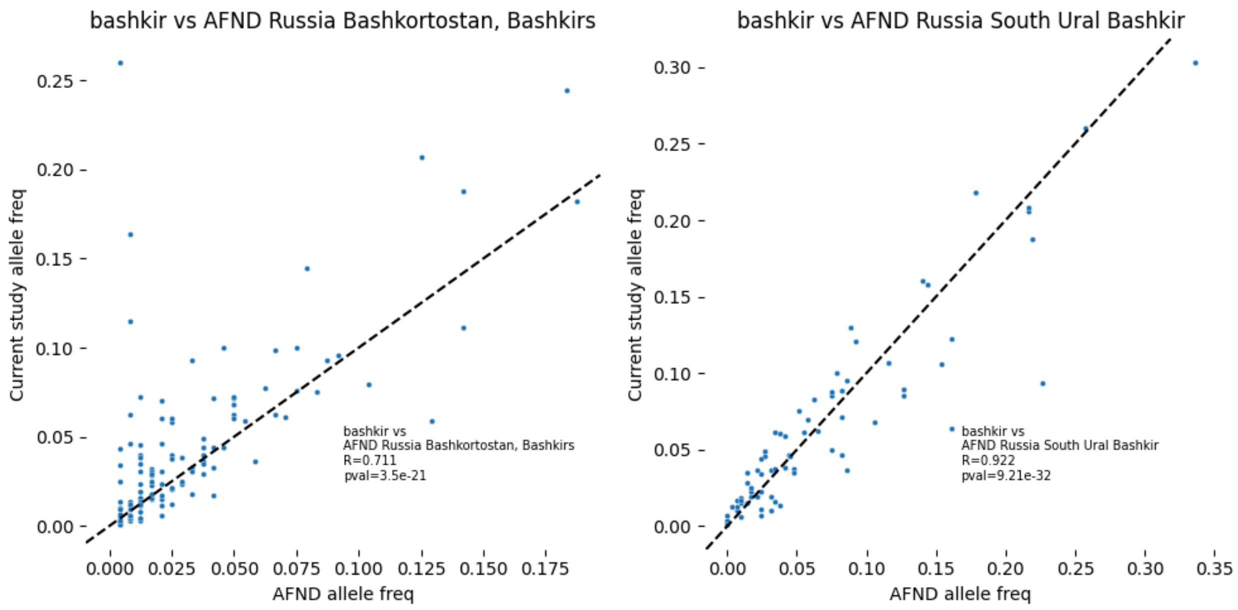

Fig S14. Allele frequency correlation of the current study cluster bashkir and the corresponding AFND datasets, with the black dashed line representing the line of perfect haplotype frequency agreement (y=x).

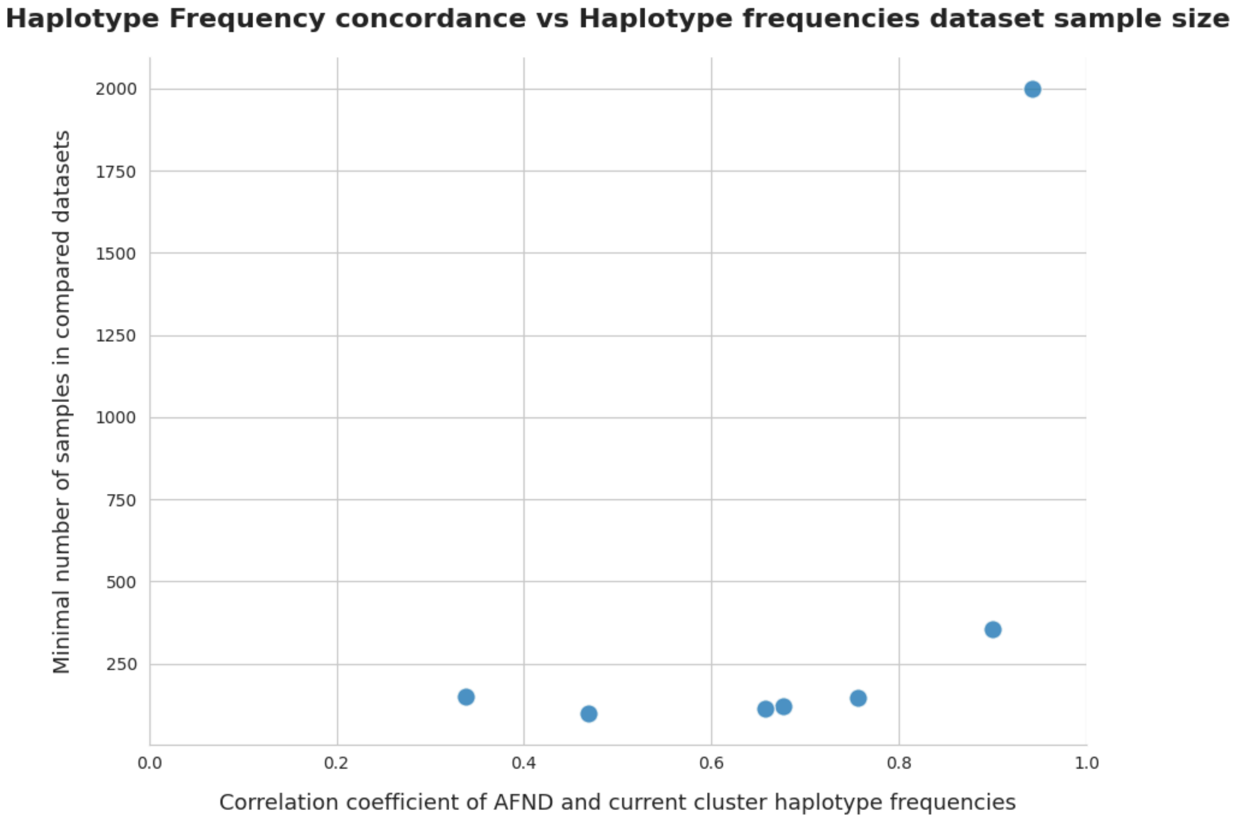

Figure S15. Relationship between sample size and concordance of haplotype frequency estimates. The x-axis shows the Pearson correlation coefficient between haplotype frequencies reported in the AFND and the corresponding dataset from the current study. The y-axis represents the minimum sample size of the two datasets included in each

comparison.

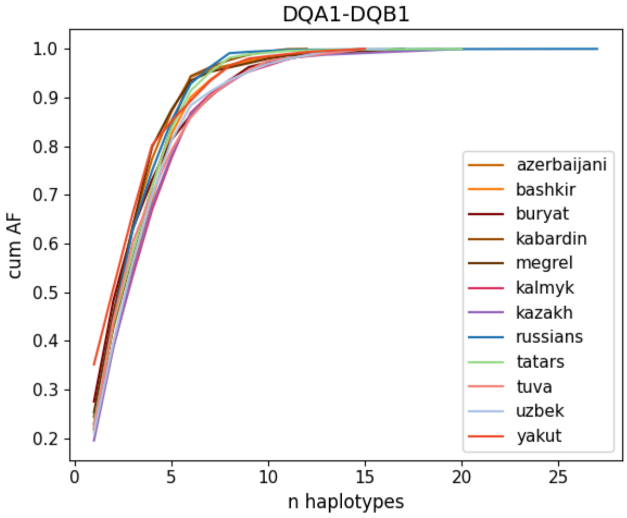

Fig. S16 Frequency saturation curves for DQA1-DQB1 haplotypes in the current study cohort.

Table S3. Summarized Pearson correlation coefficients for pairwise allele frequency comparisons between the study ethnic clusters and populations from the AFND. Values represent the mean, median, and standard deviation (SD) of correlation coefficients calculated across all valid population pairs within each cluster.

	**Mean**	**Median**	**SD**
**azerbaijani**	0.879567	0.884431	0.041635
**bashkir**	0.816571	0.816571	0.148789
**buryat**	0.832332	0.78839	0.087048
**kabardin**	0.801889	0.790426	0.062916
**megrel**	0.899475	0.929512	0.078776
**russians**	0.899241	0.983245	0.129977
**tatars**	0.80247	0.956707	0.290810
**tuva**	0.867607	0.864533	0.042541

Table S4. Population cluster pairs with significantly different distributions of T1D PRS values

combination	pval	adjusted_pval
(yakut, russians)	2.46E-21	2.24E-19
(yakut, kabardin)	2.75E-18	2.48E-16
(yakut, tatars)	6.65E-14	5.91E-12
(kazakh, kabardin)	1.55E-12	1.37E-10
(kazakh, russians)	3.62E-12	3.15E-10
(buryat, russians)	5.37E-11	4.62E-09
(buryat, kabardin)	6.58E-11	5.59E-09
(yakut, bashkir)	2.46E-10	2.06E-08
(tuva, kabardin)	3.47E-09	2.88E-07
(tuva, russians)	5.80E-09	4.75E-07
(yakut, megrel)	3.24E-08	2.63E-06
(buryat, tatars)	2.45E-07	1.96E-05
(tatars, kabardin)	3.05E-07	2.41E-05
(kazakh, tatars)	7.79E-07	6.08E-05
(uzbek, kabardin)	1.11E-06	8.56E-05
(tatars, russians)	3.58E-06	2.72E-04
(tuva, tatars)	4.16E-06	3.12E-04
(buryat, bashkir)	4.27E-06	3.16E-04
(uzbek, russians)	1.38E-05	1.01E-03
(yakut, azerbaijani)	2.03E-05	1.46E-03
(tuva, bashkir)	2.31E-05	1.64E-03
(buryat, megrel)	3.62E-05	2.53E-03
(kazakh, bashkir)	4.15E-05	2.87E-03
(kalmyk, kabardin)	9.03E-05	6.14E-03
(tuva, megrel)	9.68E-05	6.48E-03
(yakut, mansi)	1.79E-04	1.18E-02
(kalmyk, russians)	1.98E-04	1.29E-02
(bashkir, kabardin)	2.97E-04	1.90E-02
(kazakh, megrel)	3.77E-04	2.37E-02
